# Diabetic Ketoacidosis and COVID-19: A Case Series From an Inner-City Community Teaching Hospital in New York

**DOI:** 10.7759/cureus.26580

**Published:** 2022-07-05

**Authors:** Sowmya Boddhula, Satish Kumar Boddhula, Pavani Reddy Garlapati, Meet J Patel, Sunday Ekanem, Sreedhar Adapa, Vincent Fong, Swetha Balaji, Swetha Murthi, Vijay Gayam

**Affiliations:** 1 Endocrinology, Diabetes and Metabolism, Christus Good Shepherd Medical Center, Longview, USA; 2 Hospital Medicine, Christus Good Shepherd Medical Center, Long View, USA; 3 Internal Medicine, Interfaith Medical Center, Brooklyn, USA; 4 Nephrology, Adventist Medical Center, Hanford, USA; 5 Endocrinology, Diabetes and Metabolism, University of Cincinnati College of Medicine, Cincinnati, USA; 6 Internal Medicine, Balaji Hospital, Ranipet, IND; 7 Endocrinology and Diabetes, Yuma Regional Medical Center, Yuma, USA

**Keywords:** kpd, inflammatory markers, diabetes mellitus, dka, covid-19

## Abstract

Introduction: Throughout the coronavirus disease 2019 (COVID-19) pandemic, studies have repeatedly shown that COVID-19 outcomes are more severe in the elderly and those with comorbidities, with diabetes being a significant risk factor associated with more severe infection. Here, we present the clinical characteristics of 25 patients with pre-existing type 2 diabetes mellitus who presented with diabetic ketoacidosis (DKA) and COVID-19 in a community hospital in Brooklyn, New York, and identify possible predictors of mortality.

Methods: This retrospective case series recruited patients from March 1st to April 9th, 2020, with lab-confirmed COVID-19 and met DKA criteria on admission (based on American Diabetes Association diagnostic criteria for DKA).

Results: Of the 25 patients who met the inclusion criteria, 22 were African American and three were Hispanic. Common comorbidities in addition to diabetes were hypertension, obesity, coronary artery disease, and dyslipidemia. Fever, cough, myalgias, and shortness of breath were common presenting symptoms. Most patients had elevated inflammatory markers erythrocyte sedimentation rate, C-reactive protein, D-dimer, and ferritin, but higher values increased the odds of mortality. The overall survival was 64%, with those recovering having more extended hospital stays but requiring less time in the intensive care unit. At the same time, those who died were more likely to require mechanical ventilation, have an acute cardiac injury, and/or be obese. Despite numerous studies on COVID and diabetes, only a few studies described DKA.

Conclusion: This observational retrospective study illustrated that patients with diabetes are at risk of developing DKA with COVID-19 and identified some predictors of mortality. However, further studies with larger sample sizes and a control group are necessary to understand better the effects of COVID-19 on DKA and their clinical outcomes.

## Introduction

The coronavirus disease 2019 (COVID-19), caused by a novel coronavirus, a severe acute respiratory syndrome coronavirus 2 (SARS-CoV-2), is a global pandemic, infecting over 539, 119,771 cases worldwide and resulting in 6,322,311 deaths [1]. The United States has the highest number of cases, around 5,637,502 on January 10, 2022, and the highest number of fatalities at 1,004,390 so far than any other country [1]. Coronaviruses are enveloped, single-stranded (ribonucleic acid) RNA viruses that belong to the Coronaviridae family. Coronaviruses that caused severe infections in humans in the past include severe acute respiratory syndrome (SARS-CoV) with a mortality rate of 10% in 2002-2003 and Middle East respiratory syndrome (MERS-CoV) with a mortality rate of 37% in 2012 [2]. During the epidemics of MERS and SARS, patients with diabetes and other comorbidities were at increased risk of developing a more severe form of pneumonia. Similarly, COVID-19 is more prevalent in older patients and is also more severe in patients with comorbidities, including diabetes [3]. Moreover, inadequate glycemic control is associated with increased mortality in patients with diabetes and those infected with SARS-CoV-2. However, increased insulin resistance and hyperglycemia have also been reported in patients with COVID-19 with and without a prior diagnosis of diabetes, raising the question of increased incidence of diabetic ketoacidosis (DKA) in patients with diabetes and/or new-onset diabetes. Previous studies have focused on patients with diabetes, and reports including DKA that had small sample sizes [4-6]. Here we present the findings of 25 patients with type 2 diabetes (T2DM) hospitalized due to DKA and COVID-19. The clinical characteristics, laboratory data, imaging, and treatments are discussed to improve awareness and management of this condition in these patients with COVID-19.

## Materials and methods

Study design

This is a retrospective case series of hospitalized patients with COVID-19 infection and DKA at an inner-city teaching hospital in Downtown Brooklyn, New York. Included in our study were patients with laboratory-confirmed COVID-19 infection and who met the criteria for DKA, who were admitted and had a definitive outcome (discharged or deceased) during the data collection period. Nasopharyngeal swabs were obtained at admission, and COVID-19 was confirmed by the reverse transcription-polymerase chain reaction (RT-PCR) for SARS-CoV-2. The diagnostic criteria used for DKA were blood glucose >250 mg/dL, serum bicarbonate ≤18 mmol/L, anion gap >10, pH <7.3, the presence of ketonemia or ketonuria, and effective/calculated plasma osmolality >304 mOsm/kg. 

Ethical considerations

Ethical clearance was obtained from the hospital's IRB committee. Because our study was limited to chart review with no patient interactions, the requirement for informed consent was waived. 

Data collection

Data were collected from the time period March 1st through April 9th, 2020. Demographic, social, clinical, laboratory, and radiologic data were extracted from electronic medical records and entered into a spreadsheet. The data collection spreadsheet had inbuilt checks to flag probably incorrectly entered or inappropriate data, prompting immediate corrective actions to limit inaccuracies. Collected data for laboratory tests included blood gases, serum ketones, serum osmolality, lactic acid, complete blood count, coagulation profile including d-dimers, serum biochemical tests (including renal and liver function, creatine kinase, lactate dehydrogenase, and electrolytes), troponin, interleukin-6 (IL-6), serum ferritin, and procalcitonin among others. Imaging data included chest radiographs and electrocardiograms (EKGs) for all patients. 

Statistics

The SPSS statistics software package (IBM SPSS, version 25; IBM Corp; Armonk, NY, USA) and Graphpad Prism 8 were used for statistical analysis. Frequencies and percentages were used to summarize categorical and continuous variables. The descriptive values were expressed as median and 95% confidence intervals. Simple logistic regression was performed to describe the relationship of each variable to mortality. The total sample size was not large enough to perform multivariate logistic regression. As such, there was also no correction for multiple testing. Odds ratios (ORs) are shown with their 95% confidence interval. For non-dichotomous variables, the size of the unit increase used to calculate the OR was selected based on a range of the values in the subject population. A unit size other than one was used for much larger or much smaller ranges.

## Results

General characteristics of the patients

There were a total of 25 patients identified who met the inclusion criteria. The median age was 60 years; 11 (44%) were males and 14 (56%) were females. Of these patients, 22 (88%) were African-American, and the rest were Hispanic. The majority of these patients, 21 (84%), were admitted from home, and the remaining 4 (16%) were admitted from the nursing home. The median body mass index (BMI) was 30 kg/m^2^. The most common comorbidity identified besides diabetes was hypertension 64% (16), followed by obesity 52% (13). The other comorbidities were dyslipidemia 16% (4), coronary artery disease 20% (5), chronic kidney disease/end-stage renal disease 12% (3), asthma or chronic obstructive pulmonary disease 12% (3), and cerebrovascular disease 8% (2). Ten patients (40%) were either on angiotensin-converting enzyme inhibitors or angiotensin receptor blockers. Eight patients used oral antihyperglycemic drugs, and 19 used insulin, with two using both. Patient characteristics were broadly similar between patients who recovered and those who died except for BMI and prevalence of obesity. The median BMI of patients who died was 35 kg/m^2^ compared to 29 kg/m^2^ in patients who recovered; of those who recovered and were discharged, 5/13 (31.3%) were obese, while 8/9 (88.9%) those who died were obese (Table 1).

**Table 1:** General Characteristics General characteristics of patients are presented in total and sorted by the patient outcome. Simple logistic regressions were performed, and the OR of dying and its 95% confidence interval were shown. NC: logistic regression was not calculated due to too little variability in the study population; UTC: logistic regression was unable to be calculated due to perfection separation, i.e., one group having 0% or 100% of that variable; OR: odds ratio; BMI: body mass index; COPD: chronic obstructive pulmonary disease; CKD: chronic kidney disease; ESRD: end-stage renal disease; ACEI: angiotensin-converting enzyme inhibitors; ARB: angiotensin receptor blockers; CI: confidence interval. *CI that does not include 1.

	All (n=25)	Recovered (n=16)	Died (n=9)	Death OR (95% CI)
Age, years				
Median (95% CI)	60 (53-65)	58.5 (49-67)	61 (56-74)	1 (0.97-1.1)
BMI, kg/m^2^				
Median (95% CI)	30 (27.4-34)	29 (25.8-30.9)	35 (30-44.5)	1.5 (1.1-2.2)*
Sex, n (%)				
Male	11 (44)	5 (31.3)	6 (66.7)	4.4 (0.82-29)
Female	14 (56)	11 (68.8)	3 (33.3)	0.23 (0.035-1.2)
Ethnicity, n (%)				
Black	22 (88)	15 (93.8)	7 (77.8)	NC
Hispanic	3 (12)	1 (6.3)	2 (22.2)	NC
Admit from, n (%)				
Home	21 (84)	15 (93.8)	6 (66.7)	0.13 (0.0059-1.3)
NH	4 (16)	1 (6.3)	3 (33.3)	7.5 (0.79-170)
Comorbidity, n (%)				
Diabetes	25 (100)	16 (100)	9 (100)	UTC
Hypertension	16 (64)	10 (62.5)	6 (66.7)	1.2 (0.22-7.4)
Obesity	13 (52)	5 (31.3)	8 (88.9)	18 (2.3-376)*
Asthma	2 (8)	2 (12.5)	0 (0)	UTC
COPD	1 (4)	0 (0)	1 (11.1)	UTC
Coronary artery disease	5 (20)	3 (18.8)	2 (22.2)	1.2 (0.14-9.3)
Dyslipidemia	4 (16)	0 (0)	4 (44.4)	UTC
hx of Stroke	2 (8)	1 (6.3)	1 (11.1)	UTC
ESRD	1 (4)	1 (6.3)	0 (0)	UTC
CKD	2 (8)	1 (6.3)	1 (11.1)	UTC
Home medication, n (%)				
ACEi	3 (12)	2 (12.5)	1 (11.1)	0.88 (0.037-11)
ARB	7 (28)	4 (25)	3 (33.3)	1.5 (0.23-9.2)
ACEi or ARB	10 (40)	6 (37.5)	4 (44.4)	1.3 (0.24-7.2)
Oral antidiabetic agent	8 (32)	5 (31.3)	3 (33.3)	1.1 (0.18-6.3)
insulin	19 (76)	12 (75)	7 (77.8)	1.2 (0.18-10)

Clinical presentation on admission and triage vitals

On presentation, the vitals among these patients revealed a median temperature of 98.8 degrees Fahrenheit (°F), median respiratory rate of 20 breaths per minute, oxygen saturation of 95% (on room air/supplemental FiO_2_), median heart rate of 100 beats per minute, and median systolic blood pressure 124 mm Hg and diastolic blood pressure 74 mm Hg. The most common symptom on presentation was shortness of breath in 15 (60%) patients. The other symptoms were cough in 12 (48%), fever in 11 (44%), myalgia in 9 (36%), abdominal pain in 4 (16%), and nausea, vomiting, and diarrhea in 4 (16%) of the patients, respectively. EKGs revealed new ST-T wave changes in 2 (8%) patients and a median QTc interval of 455 milliseconds. Chest X-rays showed infiltrates in most patients, unilateral infiltrates in 6 (24%), and bilateral infiltrates in 16 (64%) patients. In our study, bilateral infiltrates were associated with an eight-fold increase in the risk of death. Only three patients (12%) did not have any infiltrates, and all three survived (Table 2).

**Table 2:** Clinical Presentation on Admission and Triage Vitals Patient vitals, signs, and symptoms on presentation are presented in total and sorted by the patient outcome. Simple logistic regressions were performed, and the OR of dying and its 95% CI are shown. For non-binary variables, the size of the unit increase used for the calculation of the OR is listed. OR: odds ratio; CI: confidence interval; SBP: systolic blood pressure; DBP: diastolic blood pressure. *CI that does not include 1.

	All n=25	Recovered n=16	Died n=9	Death OR (95%CI)	Unit size of OR
Vitals, median (95% CI)					
Temperature, °F	98.8 (98.1-100.9)	98.9 (98.1-101)	98.2 (97.5-101.7)	1 (0.66-1.5)	1
Respiratory rate, breaths/min	20 (20-22)	20 (20-23)	20 (20-24)	0.95 (0.74-1.1)	1
Heart rate, beats/min	110 (99-120)	110 (95-122)	116 (82-126)	0.89 (0.58-1.3)	10
SBP, mmHg	124 (112-135)	118 (105-135)	134 (108-146)	1.1 (0.76-1.6)	10
DBP, mmHg	74 (65-79)	74 (65-82)	76 (59-81)	0.8 (0.37-1.7)	10
O_2_ saturation, %	95 (90-96)	95 (90-98)	91 (76-96)	0.96 (0.87-1)	1
Chest X-ray, n (%)					
No infiltrates	3 (12)	3 (18.8)	0 (0)	UTC	
Unilateral infiltrates	6 (24)	5 (31.3)	1 (11.1)	0.28 (0.013-2.2)	
Bilateral infiltrates	16 (64)	8 (50)	8 (88.9)	8 (1.1-167)*	
EKG					
Corrected QT interval, ms	455 (448-477)	455 (437-477)	466 (448-507)	1.1 (0.94-1.4)	1
New ST changes, n (%)	2 (8)	1 (6.3)	1 (11.1)	1.9 (0.068-52)	
Symptom, n (%)					
Cough	12 (48)	8 (50)	4 (44.4)	0.8 (0.15-4.2)	
Myalgia	9 (36)	6 (37.5)	3 (33.3)	0.83 (0.14-4.6)	
Fever	11 (44)	7 (43.8)	4 (44.4)	1 (0.19-5.4)	
Shortness of breath	15 (60)	9 (56.3)	6 (66.7)	1.6 (0.29-9.5)	
Abdominal pain	4 (16)	3 (18.8)	1 (11.1)	0.54 (0.024-5.1)	
Nausea, vomiting, or diarrhea	4 (16)	4 (25)	0 (0)	UTC	
Altered mental state	12 (48)	8 (50)	4 (44.4)	0.8 (0.15-4.2)	

Laboratory parameters

The laboratory data revealed median hemoglobin of 14 g/dL, platelet count of 313 x 10^3^/uL, and white blood cells of 11 x 10^3^/uL. Serum electrolyte values were mainly within the reference range with median serum sodium 139 mmol/L, median serum potassium 5.3 mmol/L, and median magnesium 2.5 mg/dL, although many subjects, when corrected for hyperglycemia, were hypernatremic. As expected in patients with DKA, median glucose 513 mg/dL, anion gap 24 mEq/L, blood urea nitrogen 40 mg/dL, and serum creatinine 2.5 mg/dL were all elevated, while serum bicarbonate 16 mEq/L was decreased. Liver function tests were mainly within the reference range, with only a few patients having mildly elevated values. Differences in blood cell counts and fundamental chemistry values were not associated with a significant change in mortality risk (Table 3).

**Table 3:** Laboratory Values Laboratory values of the patients are presented in total and sorted by the patient outcome. Simple logistic regressions were performed, and the OR of dying and its 95% CI are shown. HbA1c: glycated hemoglobin; INR: international normalized ratio; aPTT: activated partial thromboplastin time; ESR: erythrocyte sedimentation rate; DKA: diabetic ketoacidosis; OR: odds ratio; CI: confidence interval. *CI that does not include 1.0. For non-binary variables, the size of the unit increase used for the calculation of the OR is listed.

Labs, median (95% CI)	All n=25	Recovered n=16	Died n=9	Death OR (95%CI)	Unit size of OR
Hemoglobin, g/dL	14 (12.8-14.8)	14 (12.2-14.9)	13.7 (10.7-14.8)	0.92 (0.67-1.2)	1
White blood cells, 10^3^/µL	11 (8.4-15.8)	10.6 (5.7-15.9)	15.5 (8.4-17.6)	1.1 (0.92-1.3)	1
Neutrophils, 10^3^/µL	9.1 (7.1-12.1)	8.9 (4.8-9.8)	12.7 (7.1-15.5)	1.1 (0.94-1.4)	1
Lymphocytes, 10^3^/µL	1 (0.8-1.4)	1.15 (0.8-1.9)	0.8 (0.6-1.7)	0.5 (0.091-1.3)	1
Platelets, 10^6^/µL	313 (249-358)	328.5 (225-411)	310 (200-350)	0.95 (0.49-1.8)	100
Serum sodium, mmol/L	139 (136-146)	139 (133-150)	142 (128-151)	1.1 (0.48-2.5)	10
Serum potassium, mmol/L	5.3 (5-5.5)	5.35 (5.1-5.5)	4.8 (3.8-5.9)	0.9 (0.44-1.8)	1
Serum bicarbonate, mEq/L	16 (12-18)	17.5 (10-18)	12 (10-18)	0.85 (0.66-1.1)	1
Anion gap, mEq/L	24 (23-25)	23.5 (23-25)	25 (20-32)	1.1 (0.94-1.4)	1
Blood urea nitrogen, mg/dL	40.4 (22.8-65)	40 (14-58.4)	65 (17.7-108)	1 (0.89-1.2)	10
Serum creatinine, mg/dL	2.51 (1.49-2.98)	2.525 (1.28-2.98)	2.51 (1.22-4.81)	1 (0.7-1.5)	1
Magnesium, mg/dL	2.5 (2-2.7)	2.2 (1.8-2.7)	2.7 (2-4.2)	2.6 (0.95-8.3)	1
Plasma glucose, mg/dL	513 (495-742)	537 (369-932)	513 (347-1065)	1 (0.76-1.4)	100
HbA1c, %	11.3 (10-12.9)	11.15 (9-12.9)	11.3 (10-15.5)	1.2 (0.86-1.8)	1
Aspartate aminotransferase, U/L	26 (19-40)	25 (15-40)	33 (14-96)	1.1 (0.94-1.5)	10
Alanine aminotransferase, U/L	24 (17-45)	26.5 (13-45)	24 (17-87)	1 (0.79-1.3)	10
Alkaline phosphatase, IU/L	103 (73-135)	97.5 (72-178)	108 (67-139)	0.94 (0.77-1)	10
Lactic acid, mmol/L	3 (2.2-4.2)	3.15 (1.5-4.9)	2.7 (1.9-4.4)	0.94 (0.53-1.6)	1
D-dimer, ng/mL	2568 (1500-4459)	1694 (800-3694)	8007 (2900-18000)	1.5 (1.1-2.4)*	1000
INR	1.19 (1.09-1.23)	1.15 (1.06-1.2)	1.28 (1.04-1.6)	1.8 (1.1-4.4)*	0.1
aPTT, seconds	31.4 (27.3-32.7)	31.7 (26.7-35.3)	30.2 (25.6-37.2)	1 (0.84-1.2)	1
ESR, mm/h	77 (69-98)	70 (60-88)	106 (80-119)	2.2 (1.3-4.9)*	10
C-reactive protein, mg/L	93 (77-177)	83.5 (63-100)	177 (130-272)	1.2 (1.1-1.4)*	10
Lactate dehydrogenase, U/L	492 (400-585)	451.5 (357-566)	585 (403-873)	1.4 (0.97-2.3)	100
Troponin, ng/mL	0.05 (0.03-0.17)	0.03 (0.01-0.07)	0.23 (0.03-0.43)	3 (1.4-10)*	0.1
Creatinine kinase, U/L	600 (300-1000)	450 (93-1518)	600 (300-5704)	0.99 (0.78-1.2)	1000
Brain natriuretic peptide, pg/mL	34.33 (16.14-70)	37.17 (10-76)	25.65 (16.14-183.1)	1.4 (0.73-3.2)	100
Interleukin-6, pg/mL	198 (95.4-283)	201.5 (77-300)	196 (36.5-1184)	1.2 (0.9-1.6)	100
Serum ferritin, ng/dL	1300 (655-2269)	874.5 (566-1461)	2800 (2269-3468)	1.3 (1.1-1.7)*	100
Procalcitonin, ng/mL	2 (0.6-5)	0.8 (0.4-3.2)	5 (0.9-25)	1.1 (0.99-1.2)	1
DKA-related values					
Plasma glucose, mg/dL	513 (495-742)	537 (369-932)	513 (347-1065)	1 (0.76-1.4)	100
pH	7.23 (7.18-7.24)	7.23 (7.14-7.24)	7.21 (7.1-7.26)	0.78 (0.21-2.9)	0.1
≤7.2, n (%)	10 (40)	7 (43.8)	3 (33.3)	0.64 (0.1-3.4)	-
Serum bicarbonate (mEq/L)	16 (12-18)	17.5 (10-18)	12 (10-18)	0.85 (0.66-1.1)	1
≤15 mEq/L, n (%)	12 (48)	6 (37.5)	6 (66.7)	3.3 (0.63-21)	-
Anion gap	24 (23-25)	23.5 (23-25)	25 (20-32)	1.1 (0.94-1.4)	1
Serum osmolality (mOsm/kg)	321 (308-330)	317 (303-336)	326 (305-354)	1.1 (0.81-1.5)	10
>304 mOsm/kg, n (%)	13 (52)	6 (37.5)	7 (77.8)	5.8 (1-49)	-

In contrast, measurements of inflammation and coagulation were markedly elevated, and more significant increases were associated with greater mortality. The median erythrocyte sedimentation rate was 70 mm/h in patients who recovered than 106 mm/h in patients who died. Similarly, median C-reactive protein was 83.5 mg/L vs. 177 mg/L, median D-dimer level was 1694 ng/mL vs. 8007 ng/mL, and median serum ferritin was 874.5 ng/dL vs. 2800 ng/dL, respectively, in patients who recovered compared to those who died (Table 3).

Parameters of diabetes and DKA

All of the patients in this case series had T2DM, and most had poor glycemic control, as indicated by a median hemoglobin A1c of 11.3%, and 23/25 patients had A1c >8.0%. Before a hospitalization, 19 (76%) were treated with insulin, six patients (24%) with oral medications, and two patients were treated with insulin and oral medications. As defined by the inclusion criteria for this study, all patients presented with elevated median plasma glucose levels and anion gap at 513 mg/dL and 24 mEq/L, respectively, and decreased median arterial pH at 7.23. Ten patients (40%) had moderate-to-severe DKA with a pH equal to lower than 7.2, and the remaining 15 (60%) patients had mild DKA with a pH between 7.2 and 7.3. The median lactic acid level was mildly elevated at 3 mmol/L, and the median serum bicarbonate level was reduced to 16 mEq/L. Serum bicarbonate was less than or equal to 15 mEq/L in 12 (48%) of the patients. All the patients had elevated serum and urine ketones. The median serum osmolality level in all patients was 321 mOsm/kg and 13 (52%) had levels >320 mOsm/kg and 12 (48%) had levels <320 mOsm/kg (Table 3).

Hospital course

DKA was managed with standard DKA protocol, including intravenous (IV) fluid resuscitation and IV insulin infusion, at least until the anion gap was closed. Other management included hydroxychloroquine in 17 (68%) of the patients, azithromycin in 22 (88%) of the patients, steroids in 9 (36%) of the patients for treatment of COVID-19 (Table 4), and supportive care with respiratory assistance and management of associated comorbidities, as directed by the treating physicians. Sixteen (64%) patients were eventually discharged when clinically stable, and nine (36%) passed away from complications. Negative virologic results were not a pre-requisite for discharge. The median length of hospital stay in all patients was seven days. The recovered patients stayed for 10 days and the patients who died stayed for an average of six days. Patients who recovered stayed in the ICU for a median of three days, while those who died were in the ICU for a median of five days. Patients who required ICU stay for five or more days had a much higher mortality risk (Table 5).

**Table 4:** Treatment Drugs The medications used to treat COVID-19 are shown. Simple logistic regression was performed on corticosteroid data, and the OR of dying and its 95% CI are shown. OR: odds ratio; CI: confidence interval; NC: logistic regression was not calculated due to too little variability in the study population; UTC: logistic regression was unable to be calculated due to perfection separation, i.e., one group having 0% or 100% of that variable.

	All n=25	Recovered n=16	Died n=9	Death OR (95%CI)
Treatment with, n (%)				
Azithromycin	22 (88)	14 (87.5)	8 (88.9)	NC
Hydroxychloroquine	17 (68)	8 (50)	9 (100)	UTC
Ceftriaxone	21 (84)	12 (75)	9 (100)	UTC
Corticosteroids	9 (36)	4 (25)	5 (55.6)	3.8 (0.68-23)

**Table 5:** Hospital Course Information about the patients' hospital course is presented in total and sorted by the patient outcome. Simple logistic regressions were performed, and the OR of dying and its 95% CI are shown. DKA: diabetic ketoacidosis; OR: odds ratio; CI: confidence interval; HD: hemodialysis. *CI that does not include 1.0. The unit size used for the calculation of the OR for the duration of hospital stay and ICU stay was one day.

	All n=25	Recovered n=16	Died n=9	Death OR (95%CI)
Outcome, n (% of total)	25 (100)	16 (64)	9 (36)	N/A
Hospital stay, days	7 (6-10)	10 (7-15)	6 (3-9)	0.78 (0.56-0.98)*
ICU duration, days	3 (3-4)	3 (2-4)	5 (3-7)	1.5 (1-2.5)
≥ 5 days, n (%)	7 (28)	2 (12.5)	5 (55.6)	8.8 (1.4-81)*
DKA resolution				
<72 h, n (%)	10 (40)	7 (43.8)	3 (33.3)	0.64 (0.1-3.4)
>72 h, n (%)	15 (60)	9 (56.3)	6 (66.7)	1.6 (0.29-9.5)
Complication, n (%)				
Mechanical ventilation	10 (40)	2 (12.5)	8 (88.9)	56 (6.2-1419)*
Acute cardiac injury	10 (40)	4 (25)	6 (66.7)	6 (1.1-41)*
Bacteremia	4 (16)	2 (12.5)	2 (22.2)	2 (0.2-20)
Shock	3 (12)	1 (6.3)	2 (22.2)	4.3 (0.35-102)
New-onset HD	3 (12)	2 (12.5)	1 (11.1)	0.88 (0.037-11)

In-hospital complications included 10 (40%) patients with acute cardiac injury (defined as troponins elevated >99% of the upper reference level), 10 (40%) patients requiring mechanical ventilation, 4 (16%) patients developing bacteremia, 3 (12%) patients developing shock requiring vasopressor support, and 3 (12%) patients requiring new-onset renal replacement therapy for acute kidney injury. Unsurprisingly, acute cardiac injury or respiratory failure requiring mechanical ventilation was associated with an increased risk of mortality (Table 5).

## Discussion

Effects of COVID-19 on DKA

DKA is a life-threatening complication of diabetes mellitus more commonly seen in those with type 1 diabetes mellitus (T1DM), whereas hyperosmolar hyperglycemic syndrome (HHS) is more common in those with T2DM. Nevertheless, there are substantial data on patients with T2DM presenting with DKA episodes [7]. Both can be triggered by infection and can present with severe hyperglycemia, dehydration, and altered mental status. The two conditions are differentiated largely by the presence of ketones and acidosis in DKA and not in HHS. The synthesis of ketones, which can act as mild acid, is inhibited by insulin, which is absent in patients with T1DM. Many patients with T2DM still retain some ability to produce endogenous insulin. Interestingly, all 25 of the patients in this case series had T2DM; some even were managed as outpatients with oral agents only without exogenous insulin, although it could be argued that this was suboptimal care, given their very elevated A1Cs. Nevertheless, the insulin dose required to prevent ketosis is much lower than the dose needed to avoid hyperglycemia, which raises the question of whether COVID-19 promotes insulinopenia. 

There is a subtype of T2DM, ketosis-prone type 2 diabetes (KPD, also known as Flatbush diabetes or type 1.5 diabetes), that describes patients with impaired insulin secretion in response to severe hyperglycemia, lacking typical autoimmune markers of type 1 disease, and with quick recovery of β-cell function after resolution of DKA [8,9]. It was initially reported primarily in Africans and African-Americans from the Flatbush neighborhood of Brooklyn, NY, but was subsequently recognized in Hispanics and other minority groups. Given the location and entirely black and Hispanic demographic of patients in this study, it is certainly possible that some of the patients described here had ketosis-prone diabetes. However, it seems unlikely that all had previously undiagnosed KPD. COVID‐19 infection has been previously associated with ketosis or ketoacidosis in those with and without diabetes [10]. The seemingly high incidence of DKA in patients with T2DM in this study further supports whether COVID-19 causes insulinopenia, thereby triggering DKA in these patients.

Both SARS-CoV-1 and SARS-CoV-2 bind to the ACE-2 receptor as part of the cell entry process. Previous studies with SARS-CoV-1 showed that ACE-2 was present on pancreatic islets and that infection was associated with hyperglycemia [11], suggesting a possible role for direct cellular damage in COVID-19, leading to acute islet-cell dysfunction, decreased insulin release, and subsequent acute hyperglycemic crisis and even ketosis. However, more recent studies showed conflicting data on whether pancreatic β-cells express the ACE-2 receptor and other factors related to SARS-CoV-2 entry [12,13]. Nevertheless, β-cell dysfunction does not necessarily require direct infection of endocrine cells. The studies agree that ductal cells and vasculature in the pancreas express ACE-2 and showed evidence of SARS-CoV-2 infection [13-16]. Effects of SARS-CoV-2 infection on β-cell function need more research. Nevertheless, we should not forget the more common factors precipitating DKA. In one study, infections were associated with 52% of cases of DKA, non-adherence with antidiabetic treatment (21%), and undiagnosed diabetes (11%) [17]. In our study, all patients had an infection. The median HbA1c was 11.3%, indicating that poorly controlled diabetes combined with COVID-19 respiratory infection was a solid contributor to precipitating DKA in this group. This provides an additional reason to emphasize the importance of glycemic control in patients with diabetes, especially during this pandemic. 

Effects of DKA on COVID-19

Elevated inflammatory markers have been associated with hyperglycemia and are increased during DKA and reduced throughout treatment. These elevated inflammatory cytokines have been implicated in the development of complications and poorer outcomes of DKA in the setting of COVID-19 infection, likely also worsening the course of the disease [18,19]. In a previous study from China comparing the clinical presentation of diabetic and non-diabetic patients with COVID-19, patients with diabetes were found to have imaging revealing more severe pneumonia, elevated inflammatory markers, and clotting abnormalities with a propensity toward thromboembolic events [20]. These are consistent with the finding of the current study, as all patients had elevated inflammatory markers, and most patients had lung infiltrates on imaging and mildly elevated coagulation parameters. Each of these was associated with an increased risk of death (Tables 2, 3). The interaction, if any, between the inflammation of DKA and inflammation of COVID-19 is unclear, but it is easy to speculate that each could contribute to the other and synergistically increase the risk of severe DKA or the risk of developing severe or critical COVID-19. Although notably, DKA resolved in all patients within 48 hours in this study, not all patients recovered from COVID-19.

Previous studies have shown that diabetes, hypertension, obesity, and cardiovascular disease are common comorbidities in patients with COVID-19 and are associated with more severe COVID-19 and increased mortality [3,21-23]. Factors previously associated with COVID-19-related deaths include older age, male sex, obesity, and diabetes [24,25], and the presence of fasting blood sugar >126 mg/dL was associated with increased in-hospital complications even in patients without a prior diagnosis of diabetes [26]. There is U-shaped distribution of BMI-related COVID-19 deaths with increased mortality in BMI <20 kg/m^2^ and >40 kg/m^2^ in patients with diabetes [25], and BMI is positively and independently associated with tracheal intubation and death within seven days of hospitalization [27]. In our present study, hypertension, obesity, coronary artery disease, and dyslipidemia were the most common co-morbid conditions after diabetes. However, only the presence of obesity significantly increased mortality risk (Table 1). Though notably, all four patients with dyslipidemia died, an odds ratio could not be calculated due to the perfect separation. 

DKA in older adults is associated with increased hospital and 30-day mortality [7]. Factors such as age >65 years, presence of fever in the first 24 hours, pH <7.2, and bicarbonate <15 mmol/L have been previously associated with increased mortality in patients with DKA [17]. However, in the current study, serum pH, serum bicarbonate, and patient age were similar among patients who survived and those who did not, so this did not predict mortality (Table 3). Complications like acute cardiac injury and respiratory failure requiring mechanical ventilation were associated with mortality. There was 36% mortality in our patients, and 40% required mechanical ventilation, which was lower than what was described in previous studies of COVID-19 patients [28,29]. Moreover, the median length of stay was 10 days for patients who survived, which is also lower than the previous reports among hospitalized patients in ICU [29]. This suggests that DKA may not be as severe as other conditions in the setting of COVID. Alternatively, it could be related to our small sample size, but it is consistent with a retrospective study from England that found reduced mortality in a small subset of COVID patients with DKA [30]. The mechanism that may be causing this is not clear, but one can speculate that the early high understanding of care for DKA patients may help improve outcomes.

Limitations

The significant limitations of this study are the sample size and lack of a control group with whom to compare as the level of detailed data was not measured in patients without COVID-19 infection. The total number of 25 patients limits the statistical power of the observations. Multivariate regression and corrections for multiple testing were not performed as they would not produce any meaningful results in such a small sample size. However, this also introduces the possibility of false hits. Therefore, the identified predictors of mortality should be regarded as hypothesis-generating and confirmed in a larger study. The inclusion of a control group of patients in this follow-up study would allow for better discrimination of the contribution of COVID vs. DKA to changes in laboratory values and the clinical presentation.

## Conclusions

In conclusion, this is a retrospective study of patients with DKA precipitated by SARS-CoV-2 infection. Patients had T2DM, poor glycemic control, and significantly elevated inflammatory markers. Increased BMI, increased inflammatory markers, acute cardiac injury, bilateral lung infiltrates, and respiratory failure requiring mechanical ventilation are potential factors associated with increased mortality. Nevertheless, the overall mortality was lower than that previously reported early in the pandemic, possibly related to the early intensive monitoring and aggressive supportive care necessary for the management of DKA patients. Further large-scale case-control studies are needed to understand better the factors contributing to DKA in patients with COVID-19 infection.
